# Supplementary material for: Competitive coordination of the dual roles of the Hedgehog co-receptor in homophilic adhesion and signal reception
Source: eLife. 2021 May 18;10:e65770. doi: 10.7554/eLife.65770 (PMC8131103; doi:10.7554/eLife.65770)
Supplement: Supplementary file 1. — The genotype of larvae from where wing discs were collected and imaged in each figure. [file elife-65770-supp1.docx]

| Figure 1A | - *w; ptc-GAL4, tub-GAL80^ts^/UAS-GMA-GFP* - *w; ptc-GAL4, tub-GAL80^ts^/+; UAS-Ihog/+* - *w; ptc-GAL4, tub-GAL80^ts^/+; UAS-IhogΔFn1/+* - *w; ptc-GAL4, tub-GAL80^ts^/+; UAS-IhogΔFn2/+* - *w; ptc-GAL4, tub-GAL80^ts^/+; UAS-Ihog^xHep^/+* |
| --- | --- |
| Figure 2 | (A) *w, hs-FLP; hs-FLP/+; actin>y+>GAL4/+; UAS-Ihog/+*  (C) *w, hs-FLP; hs-FLP/+; actin>y+>GAL4/+; UAS-IhogΔFn2/+* |
| Figure 3A | *w, hs-FLP; hs-FLP/+; actin>y+>GAL4/+; UAS-Ihog/+*  *w, hs-FLP; hs-FLP/+; actin>y+>GAL4/+; UAS-IhogΔFn1/+*  *w, hs-FLP; hs-FLP/+; actin>y+>GAL4/+; UAS-IhogΔFn2/+*  *w, hs-FLP; hs-FLP/+; actin>y+>GAL4/+; UAS-Ihog^xHep^/+* |
| Figure 3B | *w, hs-FLP; hs-FLP/+; actin>y+>GAL4/+; UAS-Ihog/+* |
| Figure 4 | (A) *w, hs-FLP; hs-FLP/+; actin>y+>GAL4, UAS-GFP/+; UAS-Ihog/+*  (B-D) *w, hs-FLP/+; P{y[+t7.7] w[+mC]=CoinFLP-LexA::GAD.GAL4}attP40/UAS-IhogΔFn2-HA; P{w[+mC]=UAS-CD4-spGFP1-10}3, P{w[+mC]=lexAop-CD4-spGFP11}3/LexAop-Ihog-RFP* |
| Figure 5 | (G) *w; ptc-GAL4, tub-GAL80^ts^/+; UAS-Ihog/UAS-GPI-YFP* |
| Supplementary Figure 1 | - *w; ptc-GAL4, tub-GAL80^ts^/+; UAS-myr-mRFP/+* - *w; ptc-GAL4, tub-GAL80^ts^/+; UAS-myr-mRFP/UAS-Ihog* - *w; ptc-GAL4, tub-GAL80^ts^/+; UAS-myr-mRFP/UAS-Ptc* - *w; ptc-GAL4, tub-GAL80^ts^/+; UAS-myr-mRFP/UAS-Hh* - *w, UAS-Dally/Y; ptc-GAL4, tub-GAL80^ts^/+; UAS-myr-mRFP/+* - *w; ptc-GAL4, tub-GAL80^ts^/+; UAS-myr-mRFP/UAS-Dlp* |
| Supplementary Figure 2 | (A) *w; ptc-GAL4, tub-GAL80^ts^/+; Dally-YFP/+*  (B) *w; ptc-GAL4, tub-GAL80^ts^/+; Dally-YFP/UAS-Ihog-RFP* |
| Supplementary Figure 7 | - *w; ptc-GAL4, tub-GAL80^ts^/+; UAS-GPI-YFP/+* - *w; ptc-GAL4, tub-GAL80^ts^/+; UAS-GPI-YFP/UAS-IhogΔFn1* - *w; ptc-GAL4, tub-GAL80^ts^/+; UAS-GPI-YFP/UAS-Ihog^xHep^* |
| Supplementary Figure 8 | (A*) w, boi/Y; ptc-GAL4, tub-GAL80^ts^/+; UAS-myr-mRFP/Tm6B*  (B*) w, boi/Y; ptc-GAL4, tub-GAL80^ts^/+; UAS-myr-mRFP/UAS-Ihog-RNAi* |

**Supplementary file 1. Complete list of *Drosophila melanogaster* genotypes used in this study.**

The genotype of larvae from where wing discs were collected and imaged in each figure.
